# *Salmonella*-Mediated Cancer Therapy: An Innovative Therapeutic Strategy

**DOI:** 10.7150/jca.32650

**Published:** 2019-08-20

**Authors:** Ze Mi, Zhi-Chao Feng, Cheng Li, Xiao Yang, Meng-Tian Ma, Peng-Fei Rong

**Affiliations:** Department of Radiology, The Third Xiangya Hospital, Central South University, Changsha, Hunan 410013, China

**Keywords:** bacterial-mediated cancer therapy, *Salmonella*, tumor microenvironment, combination therapy

## Abstract

Bacterial-mediated cancer therapy (BMCT) has become a hot topic in the area of antitumor treatment. *Salmonella* has been recommended to specifically colonize and proliferate inside tumors and even inhibit tumor growth. *Salmonella typhimurium* (*S. typhimurium*) is one of the most promising mediators, which can be easily manipulated. *S. typhimurium* has been engineered and designed as cancer-targeting therapeutics, and can be improved by combining with other therapeutic methods, e.g. chemotherapy and radiotherapy, which regulate the tumor microenvironment synergistically. In view of all these strengths, the engineered attenuated strains have significant advantages for tumor diagnosis and treatment. This treatment has also been approved by the FDA for clinical trial. In this review, we summarized the recent progress and research in the field of *Salmonella* -mediated cancer therapy.

## Introduction

Cancer has become a serious disease for modern societies 1. Every cancer patient is expected to receive an effective diagnosis and treatment of the tumor within a life time 2. Although conventional therapies, including surgery, chemotherapy and radiotherapy, remain the most common treatments for cancer, the limitations of these treatments have become increasingly apparent. Therefore, some novel strategies are needed to treat the tumor. Surgery is an effective treatment for tumors; however, relapse and metastasis may make surgery more difficult and risky in treating malignant tumors 3. The efficacy of radiotherapy depends mainly on causing DNA damage in tumor cells. Tissue oxygen levels play an important role during treatment. Studies have shown that low oxygen concentrations in hypoxic and necrotic regions are a common cause of treatment failure 4. On the other hand, the distribution of a drug is important for the therapeutic effect of chemotherapy, and the poor vasculature in tumors impairs drug delivery, especially in necrotic and hypoxic regions 5. Apparently, a single method cannot achieve a cure for a tumor, and different methods should be used in combination to compensate for the respective deficiencies, representing the inevitable development trend of tumor therapeutics.

In recent years, Bacterial-mediated cancer therapy (BMCT) has become the hotspot in recent research, and many attenuated bacteria produced by genetic engineering can target tumors and have local antitumor effects. BMCT has been investigated as therapeutic for over 100 years since the bone surgeon William B. Coley injected *Streptococcus pyogenes* into patients with inoperable bone and soft-tissue sarcomas 6. This treatment successfully shrunk the tumors and increased patient survival 7. However, the lack of progressive techniques and modalities to diagnose tumors and quantify changes in immune responses resulted in a poor understanding of the underlying mechanisms and difficulties in explaining Coley's results. With the development of medical technology, especially in genetic engineering, BMCT has again become a hot topic, and various kinds of bacteria, such as *Streptococcus*^8^, *Bifidobacterium*^9^, *Clostridium*^10^, and *S. typhimurium*^11^, ^12^.

*S. typhimurium* can grow under both aerobic and anaerobic conditions and thus has the capability to target and colonize nonhypoxic and hypoxic tumors as well as metastatic tumor regions accessible by the circulatory system. As a new antitumor treatment, *S. typhimurium* makes up for the gap in chemotherapy and radiotherapy in hypoxic and necrotic regions. *S. typhimurium* preferentially accumulates within tumors, forming tumor-to-normal tissue ratios exceeding 1000-10,000 to 1, and these microbes are the most efficient antitumor bacteria assayed in experimental models of cancer thus far 13, 14. Immunotherapy based on* Salmonella* has a specific "Trojan horse" tumor-attack feature, which has been used in treatment-resistant tumors 15-17. Why do *S. typhimurium* strains have significant targeting and tumor-suppressing abilities? This mechanism will be covered in more detail later in this article. Furthermore, *S. typhimurium* can combine with radiotherapy 18, 19 and chemotherapy 20-22. Combined *Salmonella* has already become the present research focus in the treatment of tumors. *Salmonella*-mediated antitumor therapy has the prospect to be a breakthrough to cancer therapy.

## *S. typhimurium* Laboratory Strains for Cancer Therapy

Numerous *S. typhimurium* mutant strains have been studied from the perspective of cancer treatment. *S. typhimurium* laboratory strains have been designed to reduce cytotoxic effects in normal organs, increase specific targeting for tumors and enhance antitumor abilities 23-25. Herein, we focused on the most common and recognized laboratory strains, VNP20009, A1-R, and ∆ ppGpp. A list of the most common mutations and their potential biological mechanisms in various tumors is described in Table 1. We also introduced some other strains in this review.

### VNP20009

VNP20009 is the only* Salmonella* strain to be evaluated in a phase I clinical trial in humans 26, and it has been widely studied in animal cancer models. VNP20009 is a genetically modified *S. typhimurium* strain possessing an excellent safety profile. To genetically attenuated virulence, the purI gene was deleted. To reduce septic shock potential and antibiotic susceptibility, the msbB gene was also deleted. VNP20009 keeps its phenotypic and genetic stability after multiple generations both *in vitro* and *in vivo*. VNP2009 can colonize and proliferate in tumor tissues very well. In tumor-modeling mice, VNP20009 accumulates preferentially in tumor tissues over normal organs at a ratio of >1000:1. After that experiment, VNP20009 was cleared from the blood and organs within 24 h by the immune system 27, 28. Recently, VNP20009 has been sequenced for other genes to improve tumor targeting. Aside from the known purI and msbB gene deletions, other mutations have also been found, including a 108-kb Suwwan deletion, 50 nonsynonymous SNPs, a purM deletion and a derivative ∆htrA 29, 30. More research is needed to prove their effectiveness and stability.

### AR-1

By using nitrosoguanidine mutagenesis, the AR-1 strain was developed. The AR-1 strain was recovered from this selection and identified as an arginine and leucine auxotroph 31. *S. typhimurium* AR-1 has a similar function as VNP20009. In addition to the tumors in Table 1, AR-1 also inhibited the growth of different cancers in mouse models of glioma^32^, prostate cancer^33^, pancreatic cancer^34^, and cervical cancer^35^, and inhibited metastasis 36, 37. Most of the latest research shows that the AR-1 strain can cause the progression of tumor cells in the cell cycle from G0/G1 to S/G2/M, therefore increasing their sensitivity to combinational chemotherapy 38. Many studies have demonstrated that cytotoxic chemotherapy kills only tumor cells in the S/G2/M phase, which are the minority in an established tumor, and has little effect on cancer cells in the G0/G1 phase 39, 40.

### ∆ppGpp

This avirulent *S. typhimurium* strain was designed by regulating endotoxin gene expression. The relA and SpoT double mutant *Salmonella* was defective in ppGpp synthesis and tested avirulent in extensive mouse models. Safety tests showed that the LD50 values with the mutant were 1*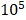 higher than those of the wild type after oral or intraperitoneal inoculation. ∆ppGpp achieved excellent cancer suppression by activating the inflammasome pathway 16, 41. This attenuated strain showed very high tumor targeting and the stimulation of regional tumor immunity. These mutant strains can induce tumor cells to secrete antitumor cytokines, such as IL-1β, IL-18, and TNF-α, thereby suppressing the proliferation of tumor models in mice. However, these cytokines have double effects on tumor cells. At low concentrations, these cytokines can stimulate and promote tumor growth. On the other hand, while normally produced at a high concentration, these cytokines can suppress tumor development 42, 43. The engineered bacteria can also express imaging reporter genes, such as Renilla and firefly luciferases 44, 45.

### Others

The three strains described above represent attenuated *S. typhimurium*, which has been very well studied with respect to cancer therapy. But beyond that, there are other mutant strains. To engineer such bacteria with better performance, we usually combine different strategies, for example SB842 (aroA, sptP)^46^, MvP728 (purD, htrA)^47^, SL (aroA)^48^-^50^, and YB1 51. In the future, we believe that an optimized balance will be achieved by the careful selection of genetic manipulations to achieve the appropriate attenuation and optimization of therapeutic benefit within the *S. typhimurium* mutant strains.

## Successful examples of Salmonella-Mediated Cancer Therapy

**Figure 1:**
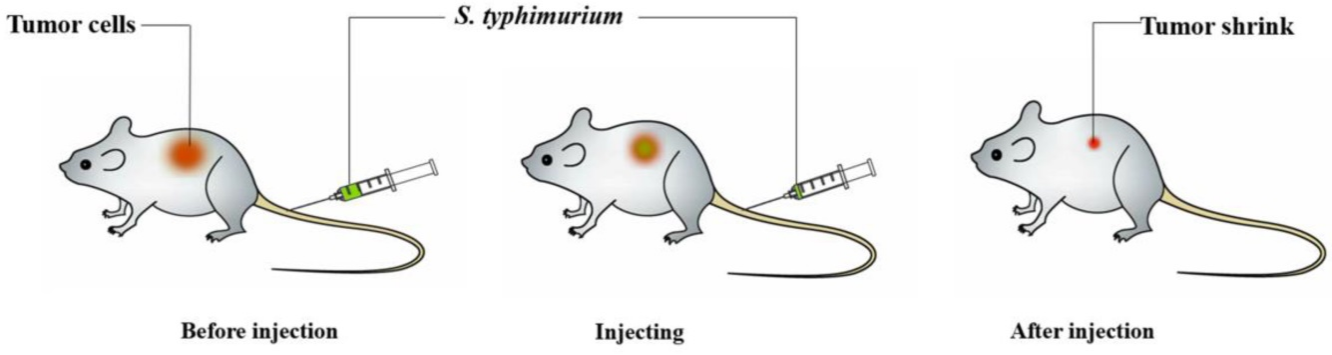
Schematic depiction of *Salmonella*-mediated cancer therapy. When administered to tumor-bearing animals, the bacteria will preferentially accumulate within tumors, especially in the nonhypoxic and hypoxic regions. Then, this treatment could obviously shrink the tumors and prolong life survival in animal models.

Among bacteria used for cancer treatment, Salmonella shows good therapeutic effects in both solid and metastatic tumors (Figure 1). Salmonella is highly regarded because of its tumor-specific localization, its ability to target various types of tumors, a fully sequenced genome, and its natural toxicity 61.

Despite the numerous advantages of *Salmonella*- mediated antitumors, the results are not satisfying in the phase I clinical trial. Single therapy using *Salmonella*, chemotherapy or radiotherapy is generally insufficient to completely cure or significantly suppress cancer growth. Therefore, nowadays, *Salmonella* combined with other therapies would be more effective in treating tumors.

### Combined with Chemotherapy Drugs

Chemotherapy for tumors is partly limited by the poor vasculature in tumors due to drug delivery impairment, especially in necrotic and hypoxic regions. *Salmonella* is capable of preferentially replicating in these poorly perfused regions. Many studies have proven that *Salmonella* combines with chemotherapeutic agents, and the results are promising 54, 62-64. The *S. typhimurium* mutant strain, VNP20009, was combined with cyclophosphamide (CTX) in a murine melanoma model. The research showed that VNP20009 significantly improved the effects of CTX treatments. The combination of CTX and VNP20009 led to a significant reduction in tumor serum vascular endothelial growth factor and capillary density compared with either treatment alone. On the other hand, combination chemotherapeutic treatment dramatically improved the number of VNP20009 within tumors when compared with bacteria treatment alone 22. Similar results were found when AR-1 combined with temozolomide or gemcitabine significantly suppressed tumor growth compared with either AR-1 alone or the chemotherapeutics alone in a mouse tumor model 58.

**Figure 2:**
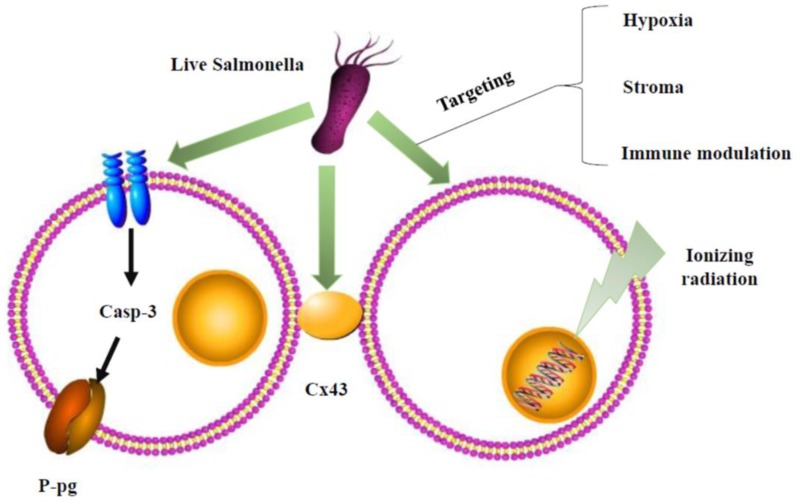
** Schematic depiction of combination therapy for Salmonella.** Salmonella could affect P-glycoprotein (P-gp) by the caspase-3 (casp-3) pathway or gap junctions, connexin 43 (Cx43), of cancer cells to enhance chemosensitivity. The underlying mechanisms of Salmonella overcome radioresistance in cancers. Salmonella changed the tumor microenvironment by hypoxia, stroma, and immune modulation to enhance radiotherapy-induced cancer cell damage.

However, the mechanism underlying this phenomenon is uncertain. Recent studies have suggested that there are three main views to explain this interaction. 1): As mentioned in the AR-1 strains, AR-1 can decoy quiescent cancer cells to cycle to S/G2/M. However, cytotoxic chemotherapy kills only cancer cells in the S/G2/M phase and has little effect on cancer cells in the G0/G1 phase. Therefore, the combination of AR-1 could sensitize tumor cells to cytotoxic chemotherapy. 2): *Salmonella* enhances chemosensitivity in tumors through connexin 43 upregulation (Figure 2). Gap junctions mediate cell communication by allowing the connexons from one cell to another. Connexons comprise six connexin (Cx) proteins. Cx43 is ubiquitous and reduced in a variety of cancer cells. Cx43 may influence the response of tumor cells to treatments by facilitating the passage of antitumor drugs or death signals between neighboring tumor cells. Many cancer cells are characterized by low expression of Cx43.* Salmonella* can activate the expression of Cx43 in melanoma cells. Cx43 can increase the susceptibility of tumor cells to chemotherapeutic-induced cell death. 3): *Salmonella* overcomes drug resistance in tumors through P-glycoprotein (P-gp) downregulation (Figure 2). Many studies have proven that P-gp is a multidrug resistance transporter 20. Recent reports have linked the overexpression of P-gp to adverse treatment effects in many cancers. This study found that the colonization of human colon tumor cell lines by *Salmonella* leads to a significant decrease and loss of protein expression in P-gp. Furthermore, these authors found the *S. typhimurium* type III secreted effector protein SipA as the key influencing factor responsible for modulating P-gp through a pathway involving caspase-3. They demonstrated that the suppression of P-gp by *Salmonella* can be achieved within cancer to enhance the efficacy and cytotoxicity of chemotherapy drugs 65.

### Combined with Radiotherapy

Radiotherapy is used to treat up to 50% of cancer patients 66. Radiotherapy for tumors works by causing DNA damage in tumor cells, particularly DNA double-strand breaking. This damage results from ionizations in the DNA that produce a radical on the DNA. DNA damage is largely dependent on the oxygen concentration in the tumor tissue. Clinical trials have proven that markedly hypoxic tumors are more radioresistant than less hypoxic tumors. Hypoxia (<1% oxygen) is a near-universal feature of tumors, making these cells particularly resistant to radiotherapy. This drawback limits treatment efficacy and is associated with increased mortality and morbidity. Moreover, preclinical studies in some tumor models have suggested that radiotherapy-induced changes may promote tumor invasion and spread in certain situations - even though decades of clinical experience have failed to show clear proof that radiotherapy promotes invasion and metastasis in patients 67. Thus, attempts to combine radiotherapy with new biologically targeted modalities were often predicated on their potential to enhance radiotherapy-induced cancer cell death rather than their potential to re-engineer biological processes within the tumor microenvironment. *Salmonella* may inhibit T-regulatory cells (T_reg_ cells), thereby increasing the CD8 T-cell to T_reg_ ratios to overcome the resistance of radiotherapy 68.

Nevertheless, *Salmonella* has unique properties that can be radioresistant. Recently, some studies proved that radiotherapy combined with an engineered *Salmonella* inhibited tumor growth compared with either radiotherapy alone or *Salmonella* alone in a mouse model of colon cancer 9. The mechanism underlying this combined treatment is not clear. A previous study indicated that the combination of *Escherichia coli*-mediated cytolytic therapy and radiotherapy inhibited tumor growth and metastasis 61. In addition, cell stroma and immune modulation also play important roles in radiotherapy resistance 4. These roles may include many mechanisms that we still do not understand (Figure 2).

### Combined with Other Therapies

**Figure 3:**
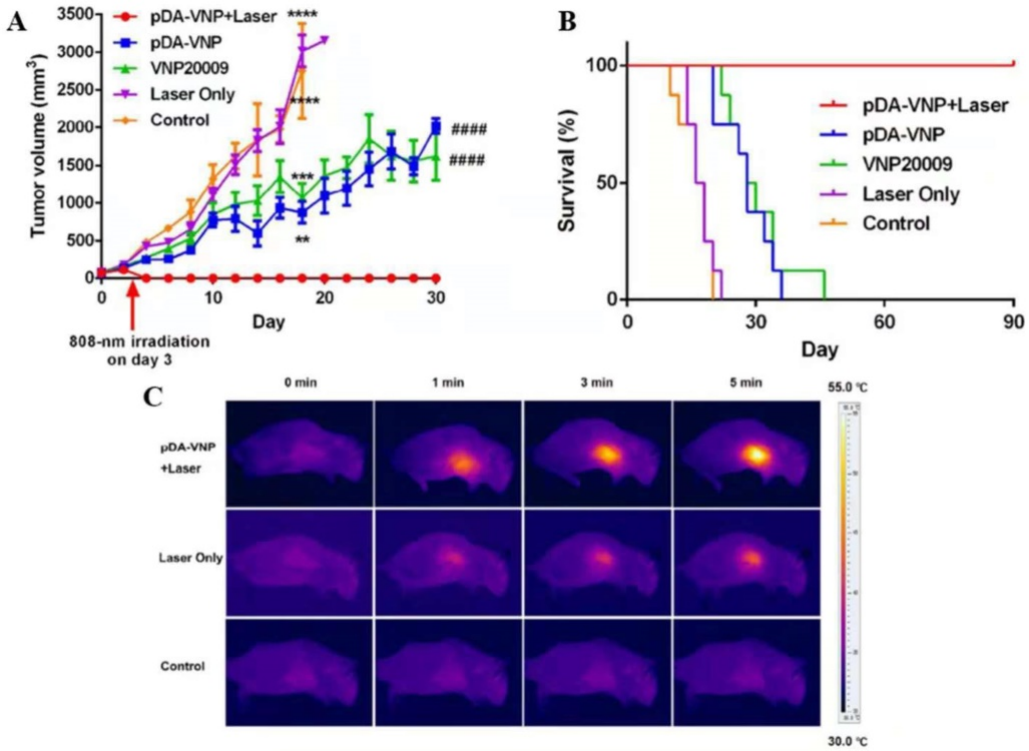
** Combination of *Salmonella* and photothermal therapy *in vivo*.** (A) Tumor volume after the indicated treatments. (B) Survival of mice after the indicated treatments. (C) Infrared thermal images of mice after the intravenous injection of pDA-VNP, followed by irradiation with a near-infrared laser (*P < 0.05; **P < 0.01; ***P < 0.001; ****P<0.0001; #P < 0.05; ##P < 0.01; ###P < 0.001; ####P < 0.0001). Reproduced with permission from 52, copyright 2018 American Chemical Society.

Given the multifactorial nature of cancer, a single treatment modality is often limited, and an increasing number of new treatments have been combined with *Salmonella* to treat tumors. The combination of the tumor-targeting ability of VNP20009 and photothermal therapy achieves enhanced specificity and antitumor effectiveness. A photothermal agent, such as melanin-like polydopamine (pDA), was coated with VNP20009 targeted to hypoxic and necrotic tumor areas. Then, a mouse model of the tumor was irradiated with a near-infrared laser, which achieved tumor targeting and tumor elimination without relapse or metastasis (Figure 3)^52^. Chinese medicine treatment of tumors is also a hot topic. Triptolide with VNP20009 could significantly enhance antitumor activity by modulating tumor angiogenesis and the host immune response 54. In contrast, combining DNA vaccines and autotransporters in attenuated Salmonella-treated tumors yielded a good effect 69, 70. We can make full use of the good targeting and the changes in the tumor microenvironment of *Salmonella*, combined with other treatments to achieve good results for tumor treatment, providing a new understanding of the strategy to improve antitumor therapy.

## Potential Cellular and Molecular mechanisms of *Salmonella*-Mediated Cancer Therapy

**Figure 4:**
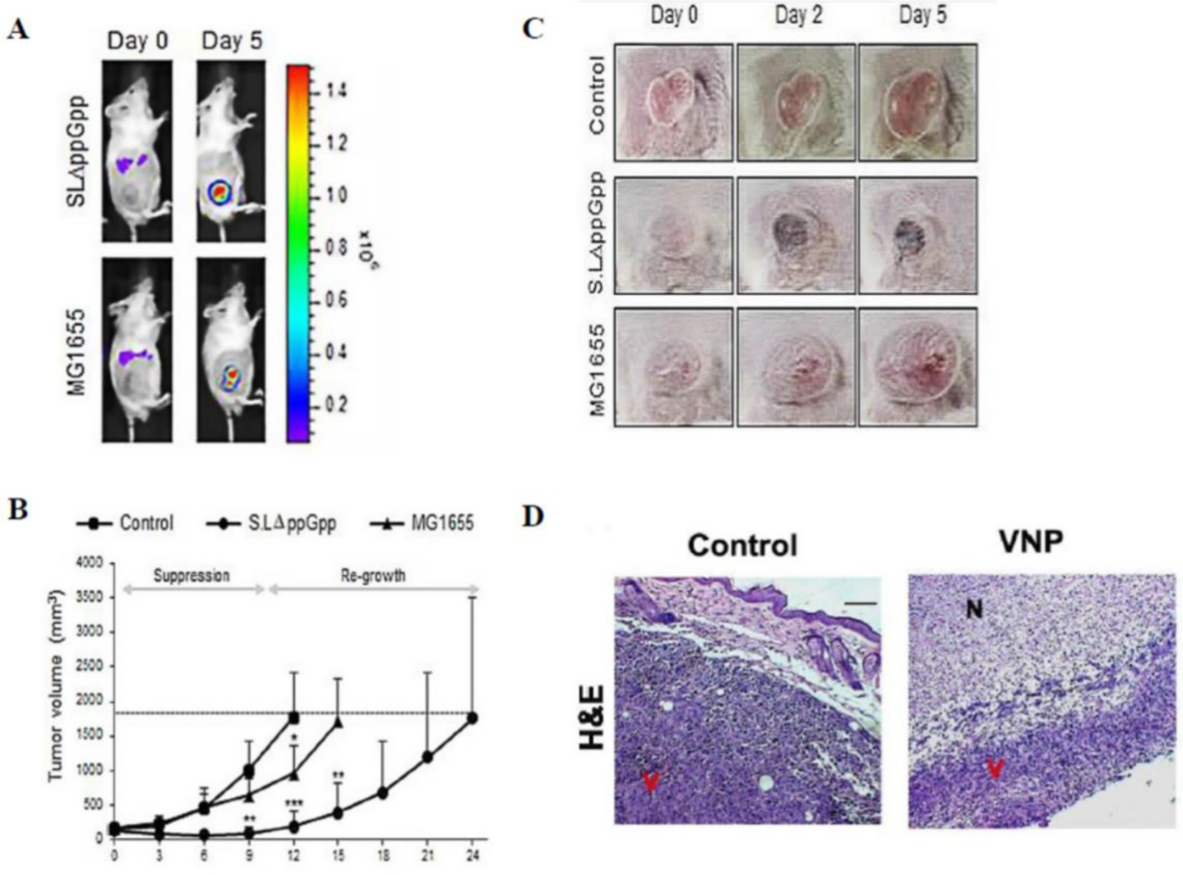
** (A-C) Systemic injection of *Salmonella* (ΔppGpp) into tumor-bearing mice induces significant growth suppression compared with the effects of *Escherichia coli* (*E. coli)* (MG1655) injection 97. (D) Systemic injection of *Salmonella* (VNP) into tumor-bearing mice induces significant growth suppression in histological examination 54.** (A) Distribution of bacteria visualized by *in vivo* bioluminescence imaging after the injection of bacteria expressing bacterial luciferase (lux). (B) The tumor volume was obviously decreased in the ΔppGpp group compared with that in the other groups. (C) The shape of the tumor was noted before (0 days) and after treatment with PBS or bacteria (2 and 5 days). (D) Histologically, the tumors treated with VNP therapy showed extensive necrosis compared with the untreated control. N: necrotic areas, V: viable tumor cells. Reproduced with permission from 54, 97, copyright 2015, 2016 Ivyspring International Publisher.

*Salmonella* has been employed as antitumor agents that are capable of preferentially assembling within tumors and inhibiting their growth (Figure 4). However, the mechanisms of *Salmonella*-mediated cancer therapy remain unclear. Some studies have found that bacteria-derived factors have an immune-stimulation effect 71. Moreover, it has been reported that *Salmonella* may have a direct antitumor effect 72, 73. Therefore, understanding Salmonella-mediated cancer therapy mechanisms will dramatically improve the therapeutics in clinical studies.

### Competitive Inhibition by *Salmonella*

**Figure 5:**
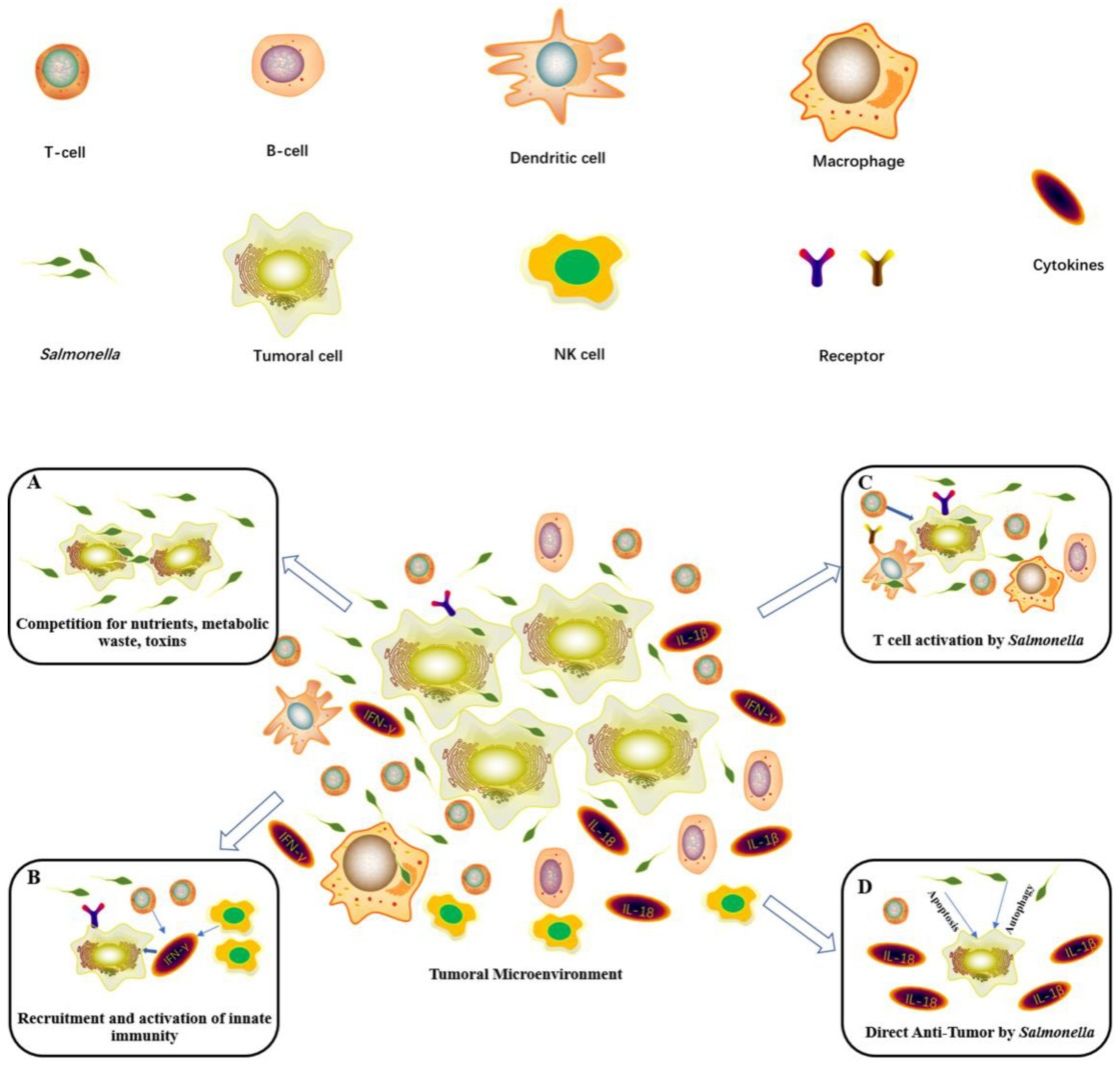
** Diagram showing the main antitumor mechanisms induced by *Salmonella*.** (A)* Salmonella* infection within the tumor microenvironment results in tumor growth inhibition and cell death. (B) *Salmonella* significantly upregulates IFN-γ and IFN-inducible chemokines to recruit NK cells and T cells to inhibit tumors. (C) *Salmonella* activates macrophages or dendritic cells by bacterial components. Then, these cells stimulate T cell activities and cytokine expression. (D) *Salmonella* can lead to the death of tumor cells by inducing apoptosis and autophagy to activate caspase or downregulating the AKT/mTOR signaling pathway. Then, it will generate IL-1β and IL-18 to antitumors.

*Salmonella* can specifically migrate to the cancer region, which is essential for *Salmonella*-mediated antitumors 23. Some studies have demonstrated many mechanisms, including the aspartate receptor, the serine receptor, and the galactose/ribose receptor on the *Salmonella* surface, which attracted *Salmonella* toward tumor cells 74. Strains lacking proper flagella constructs, active motor function, or signal transduction proteins lose the tropism for the tumor region 75. In addition, bacterial metabolism and host macrophages also play an important role in the bacterial distribution and colonization in tumor cells 76. *Salmonella* specifically accumulates and proliferates in tumor tissues over normal organs at a ratio of >1000:1 77. Therefore, large numbers of *Salmonella* colonize the tumor area. The bacterial colonization of tumor tissues deprives tumor cells of nutrients, enhances antitumor chemokines, and activates antitumor immunity, leading to cancer cell death (Figure 5A)^78^.

### Innate Immunity by *Salmonella*

*S. typhimurium* is a pathogen that causes food poisoning in humans, resulting in gastroenteritis 65. However, this pathogen can induce an immediate response mediated by the innate arm of the immune system followed by antigen-specific adaptive immunity. Some have proven that salmonella-mediated tumor inhibition relies on the induction of the innate immune response through the toll-like receptor-myeloid differentiation primary response gene signaling pathway. *Salmonella* leads to the phenotypic and functional maturation of intratumoral myeloid cells, making these cells less suppressive and hence enhancing the antitumor immune response of the host. *In vivo*, the interferon-γ (IFN) and IFN-induced chemokines CXCL9 (MIG) and CXCL10 (IP-10) showed increased expression in the tumor cells during salmonella treatment. IFN-γ- and IFN-induced chemokines may be responsible for recruiting peripheral natural killer, neutrophils, macrophages, and T cells to the tumor (Figure 5B) 79-84.

### T Cell Activation by Salmonella

**Figure 6:**
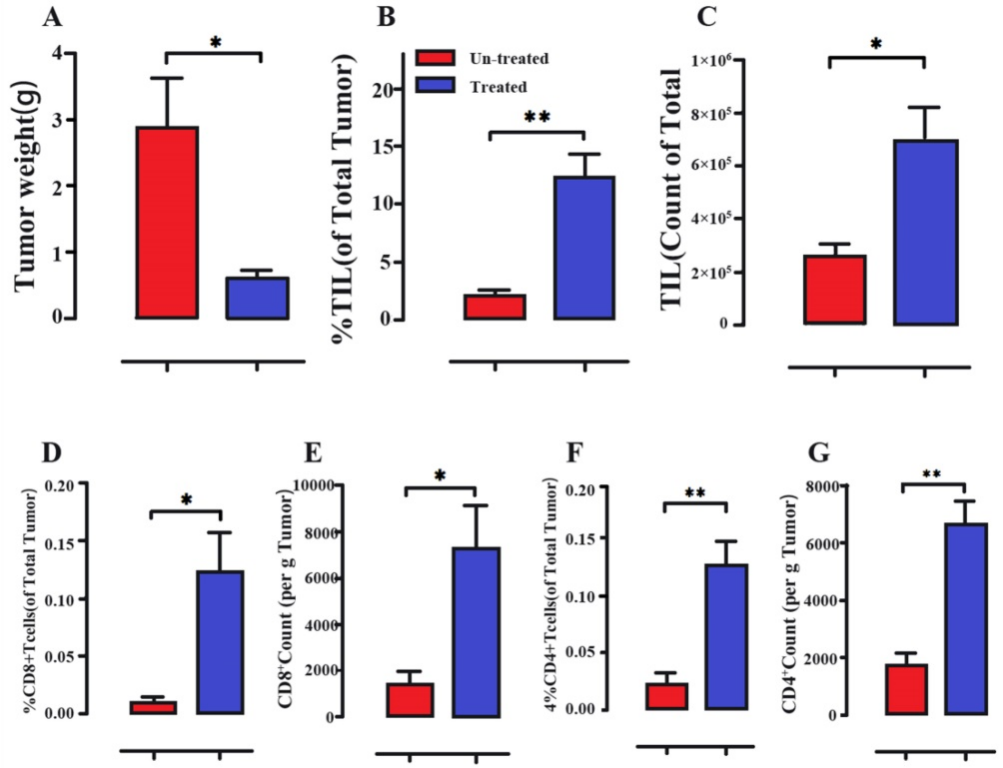
** Immune cell activation of melanoma tumors following intraperitoneal treatment with a *S. typhimurium* strain.** (A) Decreased tumor weights (B-G) *Salmonella*-treated mice obviously showed the increased percentage and absolute counts of tumor-infiltrating leukocytes (TIL), CD8+ T cells and CD4+ T cells. Reproduced with permission from 98, copyright 2018 Kaimala, Al-Sbiei, Cabral-Marques, Fernandez-Cabezudo and Al-Ramadi.

Recently, researchers proposed that T cell activation by *Salmonella* may play a key role in salmonella-mediated antitumors (Figure 6)^85^. The bacterial components, such as lipopolysaccharide, lipoteichoic acid, and flagellin, could stimulate T cells to recognize and kill tumor cells at the primary site and prevent metastasis formation 84. Salmonella replication and the lysis of tumor cells could increase the infiltration of CD4+ T cells and CD8+ T cells to enhance immune responses to tumor cells 86. Salmonella-expressing cytokines could activate T cells to modulate host immunity and inhibit tumor growth. These results proved that the stimulated T cell activities play a significant role in tumor-targeted therapy by *Salmonella* (Figure 5C)^87^, ^88^. The process of the T cell immune response could be activated by gap junctions. First, in both human and murine tumor cells, infection with *Salmonella* can induce the upregulation of connexin 43 (Cx43), a ubiquitous protein that forms gap junctions. These gap junctions transferred preprocessed antigenic peptides from the tumor cells to antigen-presenting dendritic cells (DCs), which then presented those peptides on their surfaces 89. These peptides activated CD8+ T cells against the tumor 90-92. B cells also play an important role in *Salmonella*-mediated antitumors. B cells can inhibit the dissemination of *Salmonella* to other healthy organs in *Salmonella*-mediated tumor models. In B cell-deficient mice, the bacterial loads of healthy organs were higher than those in wild-type mice 93. Inflammation from bacteremia and cytokines was found in B cell-deficient mice after *Salmonella* treatment 79, 94. However, the mechanism of this process is still unclear.

### Autophagy and Apoptosis by *Salmonella*

**Figure 7:**
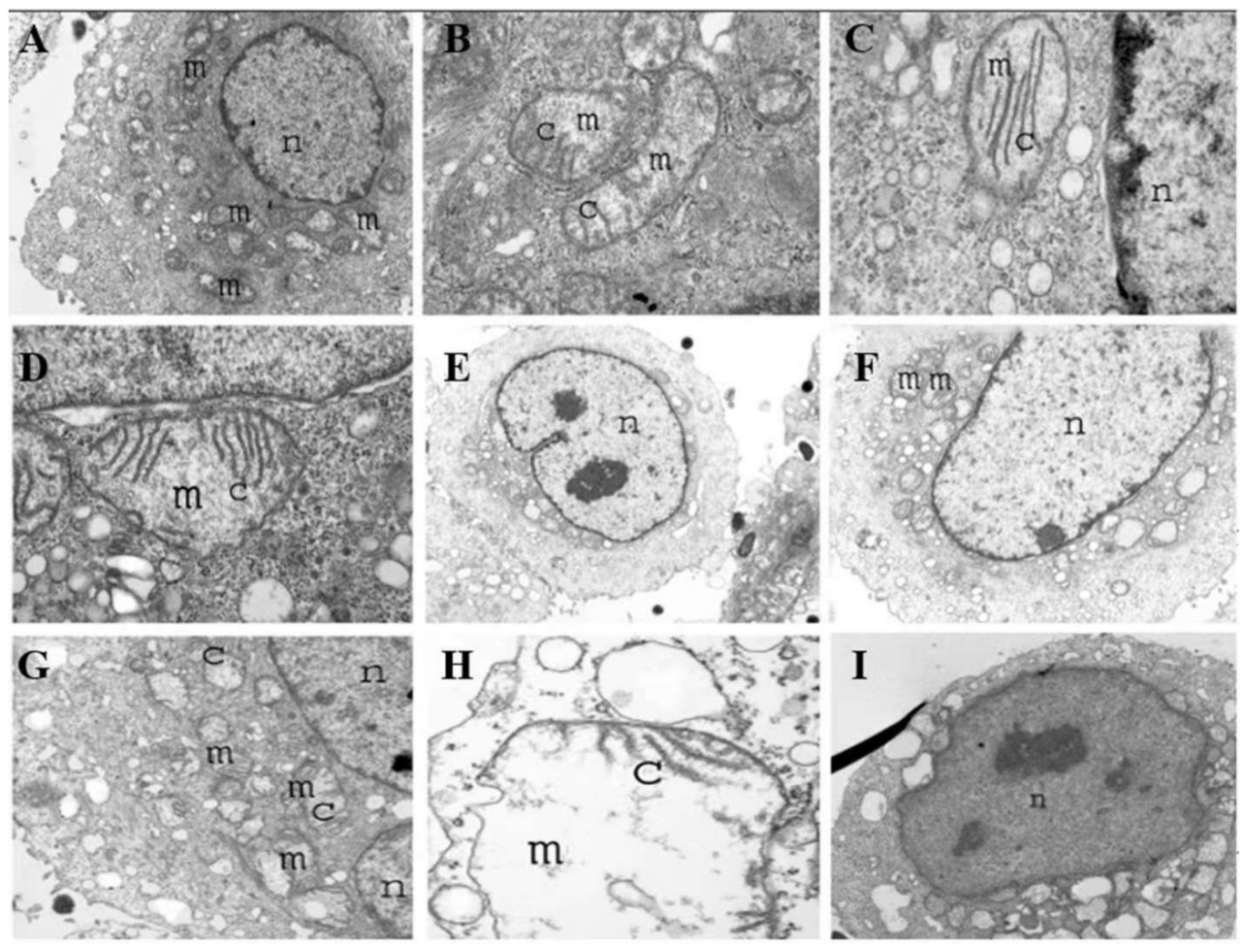
Transmission electron microscopy (TEM) of tumor cells infected with *S. typhimurium* at 1 h (A-D), 4 h (E-G), and 8 h (H, I) displaying various degrees of mitochondria destruction as a result of *Salmonella* infection. Mitochondria are shown in which cristae are destroyed gradually, leaving a large empty space and vacuole. Tumor cells eventually become apoptotic over time. Reproduced with permission from 99, copyright 2007 Cambridge University Press

*Salmonella* could induce tumor apoptosis with *Salmonella* accumulation at tumor sites (Figure 7)^73^. The induction of tumor cell apoptosis may have multiple mechanisms, including the stimulation of the immune response, secretion of toxins from bacteria and competition for nutrients. *Salmonella* may induce cell death via apoptosis and autophagic pathways by using an autophagy inhibitor and an apoptosis inhibitor.* Salmonella* can significantly decrease the factors that negatively regulate autophagy by the AKT/mTOR signaling pathway. *Salmonella*, on the other hand, could stimulate caspase-1 by inflammasomes to induce tumor cell apoptosis. Then, this enzyme will cleave pro-IL-1β and pro-IL-18 to yield active IL-1β and IL-18 to antitumors 43. Some data have suggested that infected tumor cells increased autophagy when apoptosis was blocked. *Salmonella* treatment efficiently induces both autophagy and apoptosis, which cooperate to lead to cancer cell death (Figure 5D)^95^.

In summary, the *Salmonella*-induced immune response is a complex and systemic process.* Salmonella* induces the production of proinflammatory molecules and the activation of immune cells to change the tumor microenvironment. For decades, research has focused almost entirely on the cancer cell itself, ignoring complex biological interactions between the tumor and stroma in which the cell grows - the so-called tumor microenvironment. The change in the tumor microenvironment by *Salmonella* may be a key factor for the anticancer effect.

## Conclusion and Future Perspectives

In this review, *Salmonella*-mediated antitumor therapy promoted significant tumor suppression and prolonged survival in many studies. *Salmonella*-mediated antitumor therapy has some advantages over other therapies, including proliferation, self-targeting, and ease of genetic manipulation, which are engineered attenuated strains. These characteristics make *Salmonella* an ideal and novel strategy for anticancer therapy. However, at present, *Salmonella*-mediated antitumor therapy is still not deep enough, and many problems need to be solved in practice. For example, the results achieved in the phase I clinical trial were not satisfactory. We do not know the core of the change in the tumor microenvironment by *Salmonella*. It is complicated to understand the complex interactions between *Salmonella*, inflammatory reactions and host immunity to maximize the chances of therapeutic success. Combination therapies with *Salmonella*-mediated therapy and other tumor therapies enhance the curative effects in a synergistic fashion. This strategy is a hot topic for future research. BMCT may not replace or combine all tumor treatment methods but will provide a novel treatment strategy to fight against cancer 96.

## Figures and Tables

**Table 1:** The most common *S. typhimurium* strains for targeted cancer therapy

Strains	Genotype	Target tumors	Model	Injection route	Combined with other therapies	Mechanism	In necrotic region (Yes/No)	References
VNP20009	∆purI ∆msbB	Melanoma Breast cancer Lung cancer	C57BL/6 mice 24 patients C57BL/6 mice C57BL/6 mice C57BL/6 mice BALB/c mice BALB/c mice	Intraperitoneal Intravenous Intravenous Intravenous Intravenous Intravenous Intravenous	IL-21 Photothermal Therapy Hydroxychloroquine Triptolide Antiangiogenesis peptide	IL-12 induced more NK and T cells to the tumor areas Incident light heats and kills the tumor cells Autophagy-mediated antitumors Modulating tumor angiogenesis and the host immune response Expressing the cytokine CCL21 VNP20009 carrying a Sox2 shRNA construct	Yes Yes Yes Yes Yes Yes	12 26 52 53 54 55
AR-1	arginine and leucine auxotroph	Melanoma Gastrointestinal stromal tumor Pancreatic cancer Osteosarcomas lung metastasis	Athymic nu/nu nude mice Athymic nu/nu nude mice Athymic nu/nu nude mice Athymic nu/nu nude mice Athymic nu/nu nude mice Athymic nu/nu nude mice	Intravenous Intravenous Intravenous Intravenous Intravenous Intravenous	Recombinant Methioninase (MET) Vemurafenib or temozolomide Gemcitabine Methioninase and cisplatinum	MET restriction arrests cancer cells in late S/G2 of the cell cycle AR-1 decoys quiescent tumor cells from G0/G1 to S/G2/M phase AR-1 decoys quiescent tumor cells from G0/G1 to S/G2/M phase AR-1 promotes tumoricidal CD8+ T cell tumor infiltration A1-R decoys chemoresistant quiescent cells to cycle	Yes Yes Yes Yes Yes Yes	56 21 57 58 59 60
∆ppGpp	∆relA ∆SpoT	Colon adenocarcinoma	BALB/c mice BALB/c mice	Intravenous Intravenous	Radiotherapy	*Salmonella* suppresses tumors via interleukin-1β	Yes Yes	42 18
